# Moving towards Gel for Fish Feeding: Focus on Functional Properties and Its Acceptance

**DOI:** 10.3390/gels9040305

**Published:** 2023-04-05

**Authors:** Jham Lal, Pradyut Biswas, Soibam Khogen Singh, Reshmi Debbarma, Naresh Kumar Mehta, Suparna Deb, Sanjeev Sharma, Gusheinzed Waikhom, Arun Bhai Patel

**Affiliations:** 1 Department of Aquaculture, College of Fisheries, Central Agricultural University, Lembucherra 799210, Tripura, Indiadebbarmareshmi06@gmail.com (R.D.);; 2 Department of Fish Processing Technology and Engineering, College of Fisheries, Central Agricultural University, Lembucherra 799210, Tripura, India; nareshfishco@gmail.com (N.K.M.);

**Keywords:** gel feed, gelling agents, physical properties, feed acceptability, gel stability

## Abstract

To resurrect and establish a low-impact aquaculture practice, gel-based feed applications hold promise. Gel feed is viscoelastic, nutrient-dense, hard, flexible, and appealing, and can be moulded into appealing shapes to ensure rapid acceptance by fish. The purpose of this research is to create a suitable gel feed using various gelling agents and to evaluate its properties and acceptance by a model fish, *Pethia conchonius* (rosy barb). Three gelling agents, viz. starch, calcium lactate and pectin, were included at 2%, 5%, and 8% in a fish-muscle-based diet. The physical properties of gel feed were standardized using texture profile analysis, sinking velocity, water and gel stability, water holding capacity, proximate composition, and colour. The lowest levels of nutrient leaching protein (0.57 ± 0.15%) and lipid (14.3 ± 14.30%) were observed up to 24 h in the underwater column. The highest score for overall physical and acceptance characteristics was noted for the 5% calcium lactate-based gel feed. Furthermore, a 20-day acceptance feeding experiment was conducted using 5% calcium lactate to examine its suitability as fish feed. The results indicate a better acceptability (3.55 ± 0.19%) and water stability (−2.5 ± 2.5%) of the gel feed compared to the control, with an improvement in nutrient losses. Overall, the study provides an insight into the application of gel-based diets for ornamental fish rearing, besides ensuring an efficient nutrient uptake and minimal leaching to establish a clean aquatic environment.

## 1. Introduction

Aquaculture is one of the fastest-growing animal protein production sectors, growing in volume by 7.7% annually since the 1980s [1] to make up for the consistency of fishery production over the past ten years. In fact, since 2015, the primary source of fish for human consumption has been aquaculture. This industry supplied 53% of fish in 2018, a share that is expected to rise over time as part of the strategy to provide enough food and protein to feed more than 9 billion people by 2050 [2].

Feed quality is significantly influenced by the physical characteristics of the feed, particularly its water stability [3]. Sinking granules dissolve quickly in water, lowering the quality of the aquatic environment as well as fish performance because of the oxygen-consuming biological degradation of uneaten feed [4]. Additionally, as uneaten feed is a significant source of waste generated within freshwater aquaculture, improving feeding and feed utilization stands as the only option to minimize the ecological threats to fish farming [5]. Feed accounts for 65–95% of the environmental effects of animal products leaving a farm [6]. Standing at this crossroads, the embodied intensification dimensions and the linked ecological impacts of aquaculture practice are notable, and alternative sustainability measures need to be put forth. One such example is the leaching of nitrogenous waste from aquaculture set-ups that impose negative impacts on the environment [7,8]. Another important nutrient to be concerned about is phosphorus, which is leached into the water from feed and excreta. Improper feed quality and feeding techniques contribute significantly to the environmental consequences of coastal and open-water farming techniques. One novel approach that can resolve such problematic scenarios of nutrient leaching is the application of gel-based feeds in aquaculture. Gels are transitional materials with mechanical rigidity [9]. Gels are a kind of colloid consisting of a solid three-dimensional structure that encases a liquid phase. A gel is a hierarchical structure with at least two components that exhibits solid-like mechanical properties, with the dispersed component and dispersion medium spreading evenly throughout the system [10]. Gel feeds that are nutrient-dense, hard, and flexible are made in animal-attractive shapes to ensure feed acceptance in a short time [11]. This aquatic food proportion can be used for any eatable fish food ingredient in powdered form. In general, using an aquatic food ingredient with a high protein concentration is desirable. Meat meal, crustaceans, maize meal, dried flies, fish meal, oyster meal, crab meal, wheat germ meal, oats, and other ingredients are some of the ingredients which can be used to make consumable feed. Typically, the composition contains the edible food ingredient in amounts ranging from 80 to 92% of the composition’s total weight. The edible food ingredient is moreover present as a fine powder. The application of gel feed for fishes provides a novel approach for nutrient delivery that can remain in culture tanks for at least 1–2 days without leaching to the environment. Overall, this enhances the animal performance through effective nutrient delivery, concomitant with better water quality [12]. Therefore, the application of gel-based feed harbours potential in all aquaculture set-ups, ranging from small confined aquariums in households to commercial farming.

Looking at the above, it necessitates researchers to find innovative ways to make aquaculture development more ecologically friendly in all systems, including home aquaria. Enhancing nutrient use efficiency and growth performance is a key strategy for reducing the environmental impact of aquaculture. However, novel techniques for feed formulation must be employed because feed has a significant impact on the environmental effects of aquaculture production. It is possible to reduce the environmental impacts of aquaculture by designing feeds with fewer environmental impacts, as the composition of feeds has a significant impact on their environmental friendliness. There is a clear need for the standardization of an experimental protocol to measure the leaching losses of water-soluble compounds in fish feeds and the development of simple, accurate methodologies for quantifying fish feed intake.

The rosy barb, *Pethia conchonius* is an important, highly traded, and colourful ornamental fish belonging to the Cyprinidae family. It is indigenous to the slowly moving rivers and streams of Afghanistan, Pakistan, India, Nepal, and Bangladesh [13]. Its colour is typically yellow, but males have a stronger reddish hue than females. The rosy barb typically matures sexually around 2–4 g in weight or around 6–7 weeks of age. The chromatophores of this organism have carotenoids that are deposited to give them their attractive colour, and hence they are regarded as a prized fish species for ornamental trade [14]. We considered this specimen for this experiment because of its market value, and as a model species to evaluate the effect of prepared gels using different gelling agents, as keeping ornamental fish involves a clean environment to induce a better visibility of the home aquaria. In addition, an understanding of the effectiveness of gel feed to create a safe, low-impact, and eco-friendly means of feed delivery can be revealed from this study.

In fact, the potential of using gel-based feed exists in all aquaculture setups, from the smallest household aquariums to large-scale commercial farms. Recognizing these needs, this study attempts to elucidate the functional properties of gel feed formulated with different gelling agents and its acceptability to fish, using small ornamental fish as a model. According to the author’s knowledge, this is the first published study on gel feeds for use in the ornamental fish industry.

## 2. Results and Discussion

### 2.1. Gel Strength and Texture Profile Analysis of Gel Feed

**Figure 1:**
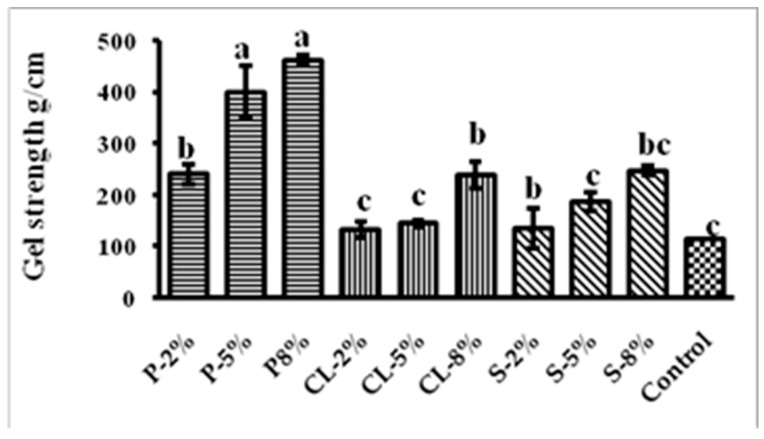
Gel strength of different gel-based feeds. Data are represented as mean ± SE. Different letters indicate significant differences (*p* < 0.05) among the experimental groups.

Gel strength is the product of breaking force and deformation [15]. A gel is formed by entrapping water molecules and other feed ingredients in the three-dimensional structure of proteins. A strong gel will leach the nutrients slowly and as result of this, will enhance the availability or utilization of nutrients. A diet supplemented with 8%pectin resulted in the highest gel strength (461.10 g/cm), while feed supplemented with 2%calcium lactate resulted in the lowest gel strength value (132.92 g/cm) (Figure 1). The gel strength of the control was 113.84 g/cm, which was shown to be considerably reduced (*p* < 0.05) when calcium lactate and starch of varied concentrations (2, 5, and 8%) were added (Figure 1). This decrease in gel strength could be attributed to increased water binding by the calcium lactate, which could result in gel structure loss and thus lower gel strength. However, across all treatments, the gel feed prepared with pectin had the highest gel strength. In an earlier work by Buda et al. (2021) [16], it was observed that fortification with 0.025% apple pectin increased the gel strength of silver carp by nearly six-fold when compared with the control, explaining the better gel strength of pectin-based gel used here.

TPA is sometimes referred to as the two-bite test because the sample is compressed twice with the appropriate probe. Table 1 displays the TPA values. Myofibrillar proteins are responsible for the majority of the strength of heat-induced gels. Camou et al. (1989) [17] discovered that increasing the heating rate reduced the gel stability. The greatest hardness value observed in our study was 31.16 N for the CL-8% based gel feed and 17.5 N for the control. When fortified with 8% CL, the highest hardness indicated better gel formation of the gel feed because it could bind more water between the three-dimensional structures of the protein than pectin and starch. The existence of ionic species and the ability to form hydrogen bonds with lactate may result in the increased hydration of myofibrillar protein. In a recent investigation, the hardness values of the jelly prepared using HPP-treated gelatine (14.19 N) and commercial gelatine (13.11 N) were found to be higher than that of the control. Furthermore, when compared with commercial gelatine, the HPP-treated gelatine produced a firmer gum. The gelatine content also influences the jelly’s firmness [18]. In the present study, the hardness of all treated gels, regardless of gelling agent concentration, increased with increasing gelling agent concentration.

In the current investigation, the adhesiveness of the gel feed was observed to range from −6.57 to −35.57 in P-8% and S-2%, respectively. These attributes are determined by the surface characteristics and the combined force of adhesion and cohesive forces [19], which are linked to the products’ molecular composition [20]. The gel feed enriched with 8% starch (S-8%) had the maximum cohesiveness value of 0.59, while the gel feed fortified with 2% pectin (P-2%) had the lowest value of 0.29. Cohesiveness is a characteristic of internal structural strength and the complexity of rupturing internal bonds, which determines how quickly a substance changes shape when subjected to mechanical force [19]. In contrast to hardness, cohesiveness reduces as gelatine concentration increases [18]. In the current investigation, the springiness of the gel feed ranged from 0.54 to 1.78 in different feeds (P-5% and CL-8%). Food’s springiness is inversely proportional to its hardness (firmness increases, elasticity decreases) [21]. As a commodity of hardness time’s cohesiveness, a secondary characteristic, gumminess, was examined. The gumminess of the gel feed with CL-8% gelling agent was 1810.53 N, which is greater than the value of 540.50 N reported for P-8%. Furthermore, the highest chewiness of the gel feed was observed for CL-8%, which was greater than the value of 334.19 for P-8%. Previous studies have shown that when a product’s hardness increases, so does its gumminess [20], since firmer jellies require more energy to breakdown into a state suitable for consumption [18,21]. Chewiness is an important texture quality of a jelly product, which reflects the strength required to masticate a long-lasting feed into a state able to be swallowed [22].

### 2.2. Colour Characteristics of Gel Feed

Gel feed colour characteristics are important prerequisites for nutrient utilization. Gel feed quantitative colour measurement parameters such as the lightness (L*), redness/greenness (a*), and yellowness/blueness (b*) of the gel feed, resulting indifferent gelling agents with different concentrations, are presented in Table 2. Feed colour may have an important influence on feed acceptance. No variations were observed in the acceptance of red, brown, or yellow diet particles when a hard pelleted diet was fed to walleye fry [23]; however, acceptance of that diet may have been limited due to diet texture. In the present study, the lightness value of the gel feed ranged from 53.6 to 63.7, where the maximum lightness was found in 2% pectin and the minimum in 8% starch. The highest redness/greenness value, 34.8, was found in CL-5% and the lowest value, 21.7, in the control on the surface region of the gel feed, whereas the yellowness value ranged from 30.9 to 39.5, where the maximum yellowness/blueness was found in CL-5% and the minimum in the control feed. The 5% pectin was more colourful in key scores such as L, a* and b*.The maximum whiteness value, 46.75, was found in the control and the lowest value, 30.83, was found in S-8%. There is a significant (*p <* 0.05) difference among the treatment and control groups. In the past, Masterson and Garling (1986) [24] established that feed colour would improve the feed acceptance and survival of walleye fingerlings fed with a soft semi-purified diet. Increasing the difference between both the food colour and the backdrop would aid in feed identification via fishes and, hence, increase feeding performance in cultivation circumstances [25]. The enhanced visual identification of feed items is linked to several parameters. This includes the light intensity and background colour of culture-rearing components [26]. Strand et al. (2007) [27] reported that suitable backdrop colour selection in the cultivation system will increase development and survival percentages in aquaculture species by increasing food acceptability for larval Eurasian perch, *Perca fluviatilis.*

### 2.3. Physical Characteristics of Gel Feed in the Water Column

**Figure 2:**
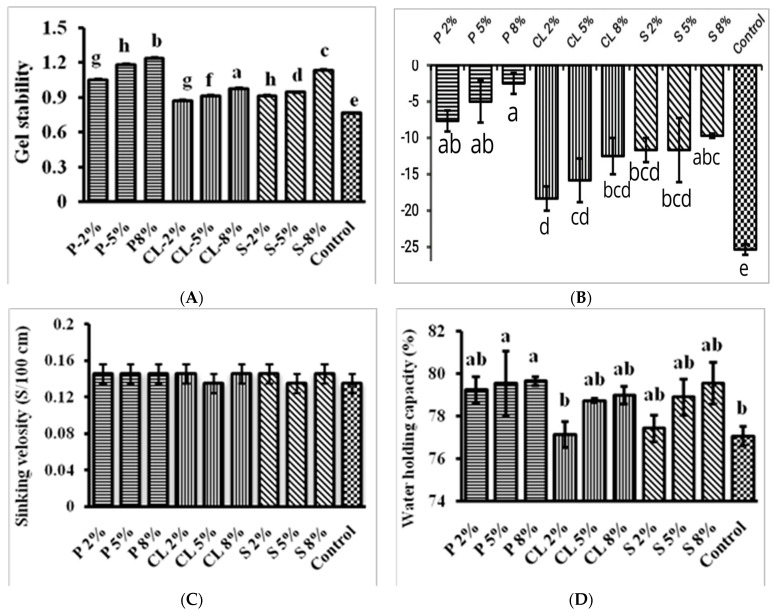
Physical properties of gel feed; (**A**) gel stability; (**B**) water stability; (**C**) sinking velocity; (**D**) water-holding capacity. Data are represented as mean ± SE. Different letters indicate statistically significant differences (*p* < 0.05) among the experimental groups.

The physical properties of all of the experimental diets are presented in Figure 2. A present study on the physical integrity of dry-formulated diets found that when immersed in running water, the dried diets degraded 10% of their dry weight basis per 10 min [28,29]. The water stability of gel feed depends on the gel strength of feed as well as gel stability. Gel strength is determined by the concentration of gelling agents as well as differences between gelling agents. Three gelling agents were used in this study to make a gel-based feed with 2%, 5%, and 8% gelling agents in feed. The current study found that 8% pectin concentration gel-based feed was more water-stable than other treatment and control groups. Starches in feeds contribute to food enlargement and gel formation, both of which have a significant impact on water stability [30]. In general, the optimum proportion of lipids improves feed texture; however, a greater lipid concentration may interact with overall binding efficiency and reduce feed stability [31,32]. In comparison with certain other gel feeds, CL-8% was linked to higher concentrations of calcium lactate (−2.50 ± 2.50) and lower concentrations of control gel feed (−33.33 ± 5.77). This may help to explain why the control feed experienced greater dry matter losses than other feed. Nonetheless, all feeds demonstrated comparable dry matter retention with substantial variations over the 24 h immersion, indicating high stability and adequate levels of all feeds relative to the control feed throughout the duration of the study. This phenomenon is likely due to the protein-rich content of fish muscle, corn flour, and the gelling agents that give gel feed its binding properties. According to Bohrer (2019), protein is crucial for hydration and dissolution, interface qualities (emulsification and foaming), taste attachment, viscosity, gel formation, texturization, and dough development [33]. As a result of the uneaten feed’s quick biological degradation, which consumes oxygen, sinking pellets have a negative impact on the water quality and fish production. In the present study, the sinking velocity of gel feed ranged from 0.13 to 0.14 in different gel feeds. These feeds remained in the water column for more than one day without nutrient leaching or feed degradation. Because of the high water content, as well as the moist type of feed, the sinking velocity of the feed is mostly determined by the visual interpretation of the feed by the fish. When sinking pellets are used in aquaculture, they quickly degrade, releasing nutrients into the environment and preventing fish from consuming them [34].

In the present study, the water-holding capacity of gel feed was observed to range from 77.06 ± 0.79 to 79.66 ± 0.35 between different gelling compositions of feed, with significant differences (*p* < 0.05) between the treatment and other control groups. The amino acid residue content, protein structure, surface polarity, and hydrophobocity are all intrinsic properties that influence a dietary protein’s ability to hold water [35]. Furthermore, food-processing techniques can have a significant impact on the amount of water food can contain. Spraying a dietary and bonding agent combination into the gelation solutions or drying chamber, where particulates form upon interaction, can also produce micro-bound particles (spray beadlets).All of the above-mentioned methods are used to variable extents in the industrial production of larval micro diets. Carbohydrates, including such gums and starches, aid in the creation of the continuous phase by reacting with both the water and proteins inside the fish paste and increasing the water-holding capacity (WHC) [36]. This improves stiffness as well as gel strength [37]. Gel stability varies depending on whether the gelling agent percentage is below or over 4%. The gel’s stability can therefore be increased in two different ways: (1) by preserving the gel formed, and (2) by preventing the structure from dissolving. Aggregation induced by entropy frequently leads to the creation of a heat-reversible gel. The agglomeration of gelators sometimes results in temporary interconnections created through physical crossings or results in permanent junctions created via chemical linkages (non-covalent bonds) by stiff transition points [38]. Similarly, Li et al. (2021) [39] reported that the WEFS engagement expanded from 2% to 7%, and the gel durability achieved the highest value of 93.92%. Additionally, the stability of a gel that is inserted into fractures and that significantly contributes to a stronger pressure difference was also examined [40].

### 2.4. Nutrient-Leaching Characteristics in the Water Column

The purpose of salts is to aid the solubilization of myofibrillar proteins, which first formed a seamless network before being thermally aggregated and cross-linked, and eventually developing fine three-dimensional steady structures that resulted in visco-elastic gels [9]. Lanter et al. [41] explained that it is possible to classify the gels as hydrogels, which are colloidal gels wherein the water serves as a dissolution medium. One factor affecting the gel’s thickness is that it should not attach to the feeding animals. Leaching assessments using two commercial and two treatment larvae feed demonstrated that between 18 and 42% of the proteins left the feed in just 2 min after watering [42]. Gel feed can be added to the water inside a fish tank. Within a gel-like substance, the fish food in the tank will be retained for a longer time period. In this manner, the aquarium’s fish find it easy to consume the food. Moreover, following preservation, the food will remain fresh in the gel. The degree of dry matter retention and nutrient loss during the specified immersion period was used to assess feed stability in water. Table 3 depicts nutritional leaching, including protein and lipids. The lowest lipid leaching was observed in P-8% and CL-5%, where only 14.3–16.7% lipid losses were observed after 24 h of water immersion. Similarly, the rate of protein leaching throughout a 24 h immersion period resulted in a maximum loss of 1.7 ± 0.6% of protein in control groups. This study concurred with that of Aaqillah-Amr et al. (2022) [43], who found that the percentage of water absorbed by the feed increased with immersion time and nutrient content. Furthermore, increased lipid inclusion causes the rapid declination of water stability and nutrient loss due to leaching. This would imply that nutrient retention in all experimental feeds was minimal during the first 6 h of immersion, which ensured optimal timing because mud crabs ingested the feeds during the first hour of immersion. Aaqillah-Amr et al. (2022) [43] investigated the nutrient leaching of semi-moist feed and found a greater percentage of dry matter nutrient losses, 87.54–88.81%, from semi-moist fish feed at 360 min of water immersion. Semi-moist feed is also more water-stable and has less nutrient leaching than other designed fish feeds. The gel maintains the feed in an appetizing state, allowing the fish to devour it at their leisure without the risk of spoilage, even after it has been in the aquarium for a comprehensive period [12]. When provided with feed material in salt water, the pellet-like product is normally stable for three to five days prior to decomposition. If employed in freshwater, the gel will often float to the surface for around four days, depending on the water’s temperature, the pace of fermentation, and the reduction in specific gravity. Because the gel structure is both soft and insoluble, it prevents the leaching of critical solubility factors such as vitamins by encasing them in a material that is both soft enough for the crab to eat at a rate compatible with its swallowing mode and quick enough to prevent further leaching. The preferred version has significant advantages over earlier pellet-form feed in that the dissolution of essential nutrients (especially amino acids) is significantly reduced, the threat of environmental damage is significantly reduced, and the particle size is much more suitable for shrimp, thereby minimising waste production [44]. The primary causes of water pollution from fish nutrition are an increase in biological oxygen demand, and nitrogen and phosphate deposition from uneaten feed and excrement [45]. In the current study, no pollution was created during the feeding acceptance experiment, even though the extra feed was more stable for longer periods of time, up to a day, and the feed was finally consumed by the fish after a long period of time.

### 2.5. Proximate Composition in Different Gel-Based Feeds

Table 4 shows the macronutrient contents of different gelling agents and concentrations of gel-based feed, such as moisture, proteins, lipids, ash, and nitrogen-free extract, with special reference to their nutritive value for fish. Various studies revealed that the composition of the gel feed features higher amounts of moisture and lower levels of crude protein, crude fat, and ash content [41]. In the current study, proximate analysis revealed that the control group had a greater moisture content (75.39 ± 0.45), a higher protein content (39.89 ± 0.95), a higher fat content (1.00 ± 1.0), a higher ash content (15.89 ± 0.93), and a higher level of nitrogen-free extract (52.61 ± 0.68). This nutritional quality is comparable to that found in the study of Lanter et al. [46], who reported the proximate composition for gel feed on a wet basis as protein percentage 2–25%, carbohydrate percentage 3–40%, crude fat percentage 0–10%, fibre percentage 2%, and moisture content 25–90%. Similarly, Aaqillah-Amr et al. (2022) [43] reported the proximate composition of different lipid levels of semi-moist feed on a dry weight basis. The crude protein content of different treatments ranged from 42.04 to 42.89%, the crude lipid content ranged from 6.67 to 12.73%, and the ash content ranged from 8.09 to 8.56%. Food lipids are the principal dietary source that provides crabs with the energy they require to carry out vital functions such as development and reproduction [47]. Aaqillah-Amr et al. (2022) [43] investigated the effects of semi-moist diets with varying dietary fat contents on the growth and development of *Scylla olivacea*.

### 2.6. Gel Feed Acceptance by P. conchonius and Growth Performance

**Figure 3:**
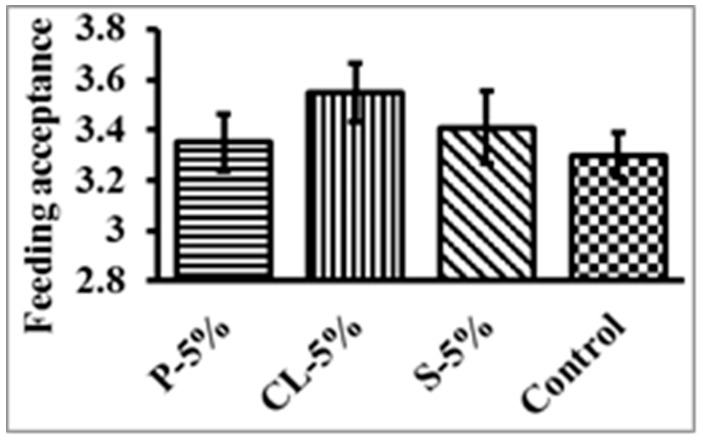
Feeding acceptance test of different types of gel feed by *Puntius conchonius*. Data are represented as mean ± SE.

**Figure 4:**
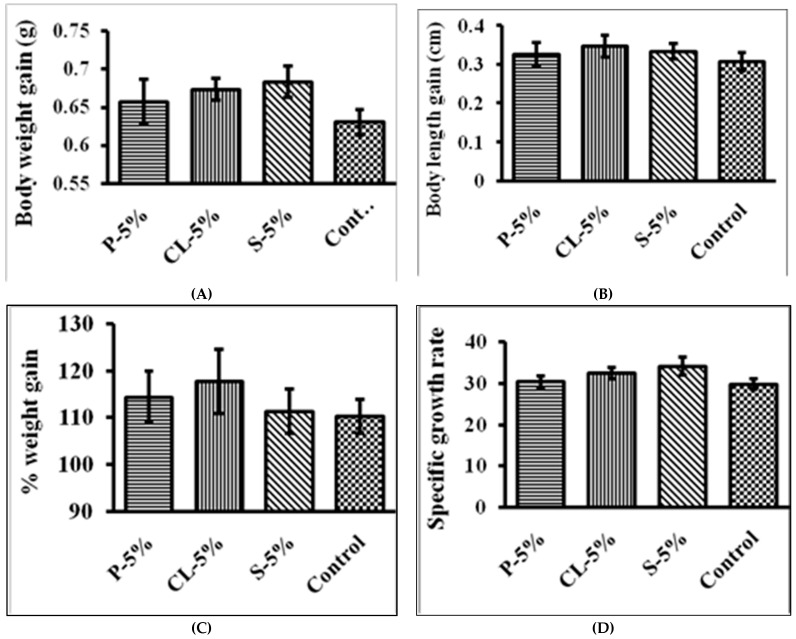
Growth performance of *P. conchonius* after 20-day feeding of gel-based feed: (**A**) body weight gain; (**B**) body length gain; (**C**) % weight gain; (**D**) specific growth rate. Data are represented as mean ± SE.

Throughout the trial, none of the treatments resulted in any deaths or signs of disease, and the rate of survival was 100%, as shown in Figure 3 and Figure 4. The rosy barb growth performance and feed acceptance test, which measured body weight gain, body length gain, specific growth rate, and feeding acceptance, revealed no statistically significant differences between the treatment and control groups. In salmon and trout farming, moist meals were utilized due to their superior acceptability, smooth surface, and lower cost in comparison to a dried diet [48]. Wet diets for cultured fish such as yellowtail and flounder no longer make sense due to the risk of water contamination from unused feed and the decline in the quality and proportion of available raw fish as a diet component. The growth performance of rosy barb and the results of the feed acceptance test, including body weight gain, body length gain, specific growth rate, and feeding acceptance, were not significantly different between the treatment feed and control groups in the current study. Aaqillah-Amr et al. (2022) [43] reported that semi-moist prepared feeds were easily absorbed by the mud crabs.

### 2.7. Water Quality Parameters

**Figure 5:**
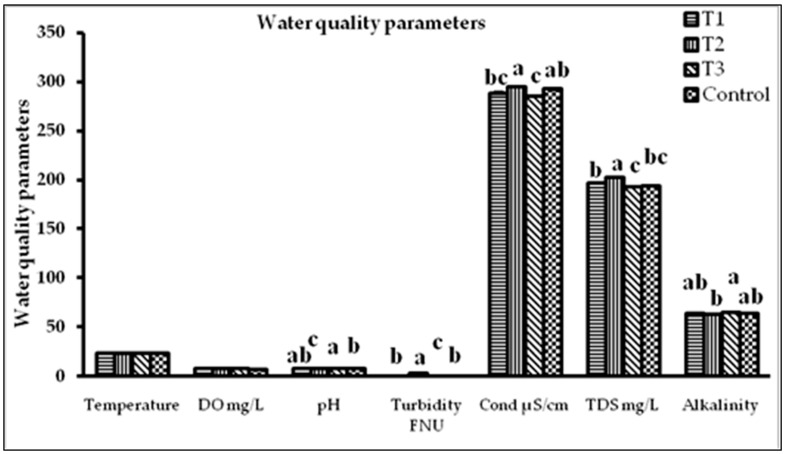
Water quality parameters in different treatment groups of rosy barb during the experimental period. Each value is expressed as mean ± standard error (M ± SE). Different letters (a,b,c) indicate significant differences determined via Duncan’s test (*p <* 0.05), *n* = 3.

The physicochemical characteristics of water in all experimental aquariums were measured on the 5th day and were determined to be within acceptable limits throughout the study period (Figure 5). Ornamental fishes are highly adaptable to cultural circumstances and capable of residence in a wide range of environments [49,50]. In the present study, the range of water variables were temperature (23.2 to 23.25 °C), DO (7.4 to 8 mg L^−1^), turbidity (0.94–1.73 mg L^−1^), pH (7.55 to 8.24), total alkalinity (62.59–64.89 mg L^−1^ as CaCO_3_), TDS (193–202.66 mg L^−1^), and conductivity (285.1–295.4 µs/cm), respectively. For the better growth, colouration, and reproduction of *P. conchonius*, Pailan et al. (2012) [51] reported the temperature range of 22–26 °C, dissolved oxygen 5.2–7.4, pH 7.2–8.1, hardness 324–380 ppm, and alkalinity 259–367 ppm. The range of the measured parameters was optimal, as compared to a study on the survival and growth of larval fish *Rasboradaniconius*, *Puntiusticto*, and *P. conchonius* [52]. Jagadeesh et al. (2015) [53] reported water quality parameters in the normal range for tropical fishes, including a temperature of 24 to 30 °C, dissolved oxygen > 5 mg L^−1^, free carbon dioxide < 5 mg L^−1^, ammonia–nitrogen < 0.1 mg L^−1^, pH 7 to 8.5, and total alkalinity 50 to 300 mg L^−1^ throughout the experimental period [54].

## 3. Conclusions

In conclusion, current research suggests that calcium lactate (CL-5%) is the predominant gelling agent for gel feed production, as demonstrated by physical qualities, biochemical contents, and feed acceptance. An analysis of feed properties revealed that CL-5% dominated in terms of feeding acceptance; however, studies on water durability and nutrient retention found that 5% calcium lactate differed considerably from some of the other feeds after immersion. The results of a comprehensive proximate composition study revealed that CL-5% was the most acceptable diet in terms of crude protein, fat, moisture, ash, and nitrogen-free extract, as well as fish reproduction and nutrient requirements. Because water is stable for more than a day, it is important to examine gel feed’s physical properties before using it in the rosy barb feed acceptance test to avoid overfeeding. The colour properties of gel feed are also a factor influencing feed vision, which is vital for feed acceptability by fish. A better colour was observed in the 5% calcium lactate. This study justifies more investigation in the form of field-based experiments to define the usefulness of gel-based diets for ornamental fish rearing and other related food fishes to ensure a cleaner and green environment.

## 4. Materials and Methods

### 4.1. Ingredients of Gel Feed

Raw ingredients were selected according to requirements for the preparation of gel feed, including fish muscle, corn flour, yeast, vitamin–mineral premix, lactogen, gelling agents, and salt.

### 4.2. Preparation of Gel Feed

The required raw ingredients were measured for the preparation of gel feed (Table 5). Fish muscle was mixed with salt, corn flour, yeast, lactogen, vitamin–mineral premix, and gelling agent (starch, calcium lactate, and pectin) at 2%, 5%, and 8% concentration, respectively. Mixed wet ingredients were taken in aluminium foil and sealed with a sealing machine. Heating is an important step for the preparation of gel feed, and so heat was provided in a water bath at 40 °C for 2.5 h. Gel feed was transferred/kept in a deep freezer for one night for the proper setting of the gel.

### 4.3. Gel Strength and Texture Profile Analysis

The texture properties of gel feed were analysed by Chandravanshi et al. (2018) [55], utilizing a texture analyser (TA-XT2 Stable Micro Systems, Surrey, UK). The gel feed was removed from the aluminium pack and cut into uniform-size pieces using a sharp blade. Samples were probed at a testing speed of 2 mm s^−1^. Texture profile analysis (TPA) was conducted utilizing an aluminium cylindrical probe (P/50) with a 50 mm diameter. Samples were compressed with testing speeds of 2 mm s^−1^ to 40% of their original height. For every treatment, the following characteristics were recorded: hardness, springiness, −1 adhesiveness, cohesiveness, and gumminess. At ambient temperature (25–27 °C), three samples from every treatment were examined.

### 4.4. Determination of Colour Characteristics of Gel Feed

A spectrocolourimeter (Colourflex EZ, Hunter Associates Laboratory, Inc., Reston, VA, USA) with a D 65/10° luminous intensity was used to measure the colour of the gel feed in triplicate. Prior to analysis, this equipment was standardized using reference tiles in black and white. Post-processing L* (lightness), a* (redness/greenness), and b* (yellowness/blueness) measurements were taken after placing a horizontal segment of gel feed measuring approximately 5 mm just above a light source. The study made use of the CIELAB (L*, a*, and b*) colour space. Whiteness was determined using the techniques mentioned Lanier et al. (1991) [56]:

Whiteness = 100 − [(100 − L*)^2^ + a*^2^ + b*^2^]^1/2^

### 4.5. Physical Properties of Gel Feed

#### 4.5.1. Evaluation of the Gel Stability of Gel-Based Feed

Gel stability after centrifugation was assessed using the process proposed by Li et al. (2021) [39]. The constituted gel food (30 g) was precisely weighed, positioned inside a 100 mL plastic centrifuge tube, and centrifuged at 3600× *g* for 5 min at 25 °C. Depending on the gel’s ability to hold water, different amounts of water and gel were separated under the influence of centrifugal force. The formula Gr = W_r_/W_i_ was used to determine the gel retention rate, where Gr (*w*/*w*) stands for the gel retention rate following samples being centrifuged, W_r_ is the gel’s residual weight after samples were centrifuged, and W_i_ is the gel’s starting weight before samples were centrifuged.

$$\mathrm{Gr}=\frac{W_{r}}{W_{i}}$$

#### 4.5.2. Water Stability

According to the methods of Das et al. (1993) [57] and Umar et al. (2013) [58], which were adapted to allow the improved replication of real-world sturgeon feed intake circumstances, the overall water stability (WS) of gel food was determined by the percentage of feed weight that was decomposed throughout 10 min under fresh water. A total of 500 mL of distilled water was transferred to a flask containing 100 g (the initial sample weight) for every feeding, and the flask was then horizontally shaken at a speed of 50 rpm. After 10 min, feeds were filtered through a 1 mm strainer on paper filters. The leftovers were filtered through water and then dried at 50 °C for 24 h before being measured (final sample weight) to determine the overall percentage of the feed that had dissolved. There were three replications for every feed’s analysis.

$$\mathrm{WS}\;(\%)=\frac{Final\;sample\;weight-Initial\;sample\;weight}{Initial\;sample\;weight}\;\times \;100$$

#### 4.5.3. Sinking Velocity (SV)

Using an adapted version of the protocol, the sinking velocity (SV) was evaluated by Das et al. (1993) [57]. There was 100 cm of water in a 105 cm rising water column. A total of 30 randomly chosen gel feed granules were placed one at a timeinto the water, and the amount of time it took for each of them to sink was precisely timed to within 0.1 s.

$$\mathrm{Sinking}\;\mathrm{velocity}\;(s/100\;\mathrm{cm})=\frac{Sinking\;time\;\left(s\right)}{Water\;column\;height\;\left(cm\right)}$$

#### 4.5.4. Water-Holding Capacity (WHC)

The water-holding capacity was determined using the method described by Barrera et al. (2002) [59]. A total of 5 g of the sample was weighed and placed between eight thicknesses of filter paper (Whatman No. 1). Components were placed at the bottom of 50 mL centrifugation tubes and then centrifuged for 15 min at 5000× *g* around 4 °C (make REMI, Mumbai, India). This gel was withdrawn and re-weighed instantly. The WHC was calculated using the following formula:

$$\mathrm{WHC}\;(\%)=\frac{W2}{W1}\;\times \;100$$

where, W1 denotes the sample’s initial weight and W2 denotes the weight of the samples after centrifugation.

### 4.6. Nutrient Leaching (Protein and Lipids)

The gel-based feeds were maintained at less than 25 °C in the water. The nutrient leaching was examined following methods of Aaqillah-Amr et al. (2022) [43] to evaluate the desired nutrient leaching at 6 h, 12 h, 18 h, and 24 h, and the feeds were taken out of the water column. The feeds were oven-dried for 72 h to retain the nutrient content for nutrient leaching analysis. The dry samples were weighed and the water stability in each feed was calculated as the ratio of recovered dry matter to dry matter of the original samples, expressed as a percentage. The proximate composition of gel-based feed was calculated after estimating the nutrient leaching. Lipid and protein analyses were carried out following methods adapted from AOAC (1995) [60]. Nutrient leaching, including protein and lipids (% dry matter basis) was calculated as follows:

$$\mathrm{Protein}\;\mathrm{or}\;\mathrm{lipid}\;(\mathrm{leaching},\%)=\frac{\left(Initial\;nutrient-final\;nutrient\;\right)}{\left(Initial\;nutrient\right)}\;\times \;100$$

### 4.7. Proximate Analysis of Gel Feed

The nutritional profile of gel feeds containing different gelling agents was analysed using the described method of AOAC (1995) [60] in the analytical lab, Department of Aquaculture, College of Fisheries, Lembucherra, Tripura. A fixed amount of gel feed was dried in a hot air oven with a temperature of 100 °C for more than 24 h until a weight change was noticed, in order to measure the moisture levels. By putting a known quantity of dried gel feed inside a silicon crucible and heating it to between 500 and 600 °C for 5–6 h, the total ash content was determined. Through semi-automated distillation and titration using the nitrogen concentration multiplied by 6.25, this protein concentration of dried gel feed samples was systematically investigated.

A thimble was used to collect one gram of the gel feed sample, which was then put in a soxhlet extraction device. The soxhlet flask’s initial weight was noted, and 150 mL of petroleum ether (boiling point 40–60 °C) was added. For eight hours, this same petroleum ether was permitted to heat up for circulation with a thimble using a siphoning procedure, with soxhlet extraction set up on the heater’s metal. Petroleum ether was removed from the flask and left to evaporate.

### 4.8. Feeding Acceptance Test of Gel Feed

#### 4.8.1. Experimental Fish

Individuals of *P. conchonius*, the rosy barb, were randomly selected and anesthetized (MS-222, 40 mg L^−1^), and the beginning length and weight were noted. The initial length ranged from 4.7 to 5 gm (4.86 ± 0.15) and weight ranged from 1.4 to 1.6 cm (1.5 ± 0.1). The fish were acclimated in aquarium tanks for 4–5 days before release into the experimental tanks; during that period, a commercial feed was given to them.

#### 4.8.2. Experimental Design and Tank Management

The feeding acceptance of four gel feed treatments was assessed in triplicate in the same tanks (all treatments were carried out in one tank, i.e., T1, T2, T3, and T4 (control)). Individual fish were randomly stocked in an aquarium (60 cm length × 30 cm width × 45 cm height) containing a total volume of 45 L of water, at the rate of 12 fish per aquarium tank, following a completely randomized design (CRD).The aquarium was divided into four compartments, distinguished by a white hard plastic wall 6 to 7 cm in height and an open water surface that enabled the fish to glide willingly between all enclosures without combining the different gel feeds. Fish were maintained on a 12:12 h light/dark cycle. To make sure that the unconsumed pellet would not disintegrate when left for an entire day in the small tanks, these same four different gel diets were evaluated for stability under water before the experiment.

To achieve a feed efficiency proportion of 7% of the body weight (BW) every day, overall feeding ratios were adjusted at 1.75 percent of the bodyweight each day for each of the four gel feeds. This total ration ensured that feed was given in excess. The feed was administered twice daily during the study (10 AM and 4 PM). The reference diet was dispensed into a single small tank, and 3 segments of the small tanks received experimental testing feeds. Every day, we carefully removed any unconsumed gel feed from each tank using a smaller mesh and weighed it. The fish usually went towards the opposite compartment once the gel feed was gathered. After nine days, these fish showed a particular preference towards one of the feeders. To account for left/right bias, the feed locations were switched in each aquarium. The experiment lasted for a total of 20 days after adding an extra day to it.

#### 4.8.3. Growth Performance and Feeding Acceptance of Gel Feed

Fish were chosen randomly from every tank and measured for their weight and length. Fish were weighed and their overall length was determined with a precision of one gram and one centimetre, respectively. Every treatment group’s average body weight, percentage weight gain, and specific growth rate were measured.

Average body weight gain (gm)

Body weight gain=Average final weight−Average initial weight

Percentage weight gain (%)

$$\mathrm{Percentage}\;\mathrm{weight}\;\mathrm{gain}=\frac{Average\;final\;weight-Average\;initial\;weight}{Average\;initial\;weight}\;\times \;100\%$$

Specific growth rate

$$\mathrm{Specific}\;\mathrm{growth}\;\mathrm{rate}/\mathrm{day}\;(\%)=\frac{(\mathrm{In}\;\mathrm{final}\;\mathrm{weight}-\mathrm{In}\;\mathrm{initial}\;\mathrm{weight})}{\mathrm{Number}\;\mathrm{of}\;\mathrm{days}}\;\times \;100$$

Feed acceptance (% of body weight/feeding session)

$$\mathrm{Feed}\;\mathrm{acceptance}=\frac{Feed\;intake\;\left(g/session\right)}{\left(Biomass\;of\;fish\;in\;the\;tank\right)}\;\times \;100$$

#### 4.8.4. Management of Water Quality Parameters

The water quality parameters were measured in the morning before the fish were fed. Water parameters were measured on every 5th day throughout all experimental tanks using the procedure outlined below, APHA (2005) [61]. The aquaculture laboratory, College of Fisheries, Lembucherra, Tripura, India was used to measure water quality parameters such as temperature, dissolved oxygen, hardness, turbidity, conductivity, total dissolved solids in accordance with Sonde (multi-parameters of water quality), pH (pH meter), and alkalinity (titration method).

### 4.9. Statistical Analysis

The data were subjected to a two-way ANOVA and one-way ANOVA, utilizing IBM SPSS Statistics (version 21.0 for Windows) to ascertain whether there were any significant differences between the different treatment groups. Then, 95% possible outcome reconciliation was performed (*p <* 0.05). The obtained data are presented as mean ± standard deviation. A post hoc Duncan’s test and descriptive comparisons test were performed after the one-way ANOVA analysis when substantial differences were discovered. Data were normalized.

## Figures and Tables

**Table 1:** Texture profile analysis of different gelling concentrations of gel-based feed.

Parameters	Concentration	Hardness (N)	Adhesiveness	Springiness	Cohesiveness	Gumminess (N)	Chewiness (N)	Resilience
Pectin	2%	28.9 ± 4 ab	−21.3 ± 13.9	0.7 ± 0.1 b	0.3 ± 0.1 f	8.4 ± 0.9 cd	5.9 ± 0.7 b	0.09 ± 0.02 cd
	5%	19.4 ± 1.8 de	−22.1 ± 1.4	0.5 ± 0.01 b	0.3 ± 0.1 f	6.3 ± 1.6 d	3.4 ± 0.9 b	0.08 ± 0.01 d
	8%	15.5 ± 0.8 e	−6.6 ± 3.7	0.61 ± 0.02 b	0.34 ± 0.02 ef	5.3 ± 0.3 d	3.3 ± 0.2 b	0.12 ± 0.0 bcd
Calcium lactate	2%	21.2 ± 2.5 cde	−10.8 ± 5.2	0.7 ± 0.03 b	0.4 ± 0.01 def	7.8 ± 0.7 cd	5.5 ± 0.5 b	0.10 ± 0.01 cd
	5%	28.5 ± 2.3 ab	−14 ± 1.4	0.8 ± 0.01 b	0.4 ± 0.0 de	11.3 ± 1 bc	9.4 ± 0.8 b	0.09 ± 0.0 cd
	8%	31.1 ± 7.7 a	−7.8 ± 2.9	1.8 ± 1.6 a	0.6 ± 0.06 ab	17.8 ± 6.1 a	32.7 ± 32.4 a	0.2 ± 0.04 a
Starch	2%	27.8 ± 5.2 abc	−35.6 ± 47.7	0.8 ± 0.04 b	0.4 ± 0.03 cd	12.3 ± 2.7 bc	9.6 ± 2.4 b	0.02 ± 0.02 b
	5%	24.5 ± 4.2 abcd	−24.2 ± 7.6	0.78 ± 0.1 b	0.5 ± 0.02 c	11.6 ± 1.8 bc	9.2 ± 2.5 b	0.02 ± 0.02 bc
	8%	22.8 ± 1 bcde	−9.5 ± 2.1	0.9 ± 0.1 b	0.6 ± 0.04 a	13.6 ± 1.4 b	11.9 ± 1.4 b	0.03 ± 0.03 a
Control	0%	17.5 ± 3.6 de	−8.8 ± 6.1	0.8 ± 0 b	0.5 ± 0.03 bc	8.8 ± 1.4 cd	6.9 ± 1 b	0.02 ± 0.02 b
*p*-value	Gelling	0.018	0.296	0.164	<0.001	<0.001	0.079	<0.001
	Conc.	0.305	0.148	0.258	<0.001	0.042	0.141	<0.001
	G × C ^1^	<0.001	0.790	0.333	0.031	<0.05	0.130	0.031

Values are means ± SD, *n* = 3 per treatment group. a–f Means in a column without a common letter differ (*p* < 0.05), as analysed by two-way ANOVA and the Duncan test. ^1^ G × C = Gelling × Concentration interaction effect.

**Table 2:** Colour analysis of different gelling agent concentrations of gel feed.

Parameters	Concentration	L* (Lightness)	a* (Redness/ Greenness)	b* (Yellowness/ Blueness)	Whiteness
Pectin	2%	63.7 ± 0.2 a	24.6 ± 0.1 e	33.5 ± 0.2 ef	44.8 ± 0.3 b
	5%	61.8 ± 0.3 c	24.9 ± 0.1 d	33.7 ± 0.5 e	43.3 ± 0.2 c
	8%	61.9 ± 0.4 c	25.02 ± 0.2 d	33.5 ± 0.7 ef	43.4 ± 0.3 c
Calcium lactate	2%	62.6 ± 0.3 b	21.9 ± 0.2 f	31 ± 0.2 g	46.7 ± 0.3 a
	5%	56.4 ± 0.2 f	34.8 ± 0.2 a	39.5 ± 0.2 a	31.7 ± 0.1 g
	8%	60.2 ± 0.2 d	28.3 ± 0.2 c	36.8 ± 0.2 c	38.9 ± 0.1 f
Starch	2%	59.6 ± 0.4 e	28.09 ± 0.1 c	35.5 ± 0.6 d	39.3 ± 0.2 e
	5%	56.01 ± 0.3 f	24.8 ± 0.4 de	32.9 ± 0.3 f	39.7 ± 0.1 d
	8%	53.6 ± 0.2 g	33.6 ± 0.2 b	38.7 ± 0.3 b	30.8 ± 0.1 h
Control	0%	62.5 ± 0.2 b	21.6 ± 0.2 f	30.9 ± 0.1 g	46.8 ± 0.02 a
*p*-value	Gelling	<0.001	<0.001	<0.001	<0.001
	Conc.	<0.001	<0.001	<0.001	<0.001
	G × C ^1^	<0.001	<0.001	<0.001	<0.001

Values are means ± SD, *n* = 3 per treatment group. a–h Means in a column without a common letter differ (*p* < 0.05), as analysed by two-way ANOVA and the DUNCAN test. ^1^ G × C = Gelling × Concentration interaction effect.

**Table 3:** Nutrient leaching of different gel-based feeds.

Parameters	Concentration	Protein				Lipid			
		6 h	12 h	18 h	24 h	6 h	12 h	18 h	24 h
Pectin	2%	0.4 ± 0.09 ab	0.6 ± 0.2 ab	0.9 ± 0.3 abcd	1.4 ± 0.06 ab	6 h	12 h	18 h	24 h
	5%	0.2 ± 0.07 abc	0.5 ± 0.03 ab	0.7 ± 0.2 bcde	1.06 ± 0.4 abcd	13.3 ± 11.5	13.3 ± 11.5	21.7 ± 2.9	28.3 ± 10.4 ab
	8%	0.1 ± 0.1 bc	0.4 ± 0.1 b	0.6 ± 0.3 cde	0.8 ± 0.4 bcd	4.2 ± 7.2	4.2 ± 7.2	13.09 ± 12.5	22.8 ± 12.8 b
Calcium lactate	2%	0.4 ± 0.2 ab	0.8 ± 0.4 a	1.2 ± 0.2 a	1.5 ± 0.2 a	4.8 ± 8.2	4.8 ± 8.2	9.5 ± 8.2	14.3 ± 14.3 b
	5%	0.2 ± 0.1 bc	0.3 ± 0.02 b	0.5 ± 0.06 e	0.6 ± 0.1 d	6.7 ± 11.5	6.7 ± 11.5	24.4 ± 21.4	24.4 ± 21.4 b
	8%	0.1 ± 0.1 c	0.3 ± 0.1 b	0.5 ± 0.1 de	0.7 ± 0.2 cd	5.5 ± 9.6	11.1 ± 9.6	16.7 ± 16.7	16.7 ± 16.7 b
Starch	2%	0.4 ± 0.2 ab	0.8 ± 0.06 a	1.02 ± 0.1 abc	1.2 ± 0.07 abc	4.8 ± 8.2	4.8 ± 8.2	9.5 ± 16.5	24.5 ± 4.3 b
	5%	0.2 ± 0.03 bc	0.3 ± 0.05 b	0.4 ± 0.2 e	0.6 ± 0.2 d	13.9 ± 12.7	13.9 ± 12.7	33.3 ± 14.4	36.1 ± 12.7 ab
	8%	0.2 ± 0.09 abc	0.4 ± 0.2 b	0.7 ± 0.4 bcde	0.7 ± 0.5 cd	6.7 ± 11.5	6.7 ± 11.5	15 ± 13.2	32.8 ± 7.5 ab
Control	0%	0.4 ± 0.09 a	0.8 ± 0.2 a	1.1 ± 0.3 ab	1.7 ± 0.6 a	12.2 ± 10.7	12.2 ± 10.7	18.9 ± 1.9	37.8 ± 3.8 ab
*p*-value	Gelling	0.791	0.957	0.931	0.907	0.528	0.697	0.337	0.018
	Conc.	0.004	<0.001	<0.05	0.122	0.454	0.617	0.286	0.946
	G × C ^1^	0.899	0.389	0.660	0.401	0.921	0.710	0.755	0.512

Values are means ± SD, *n* = 3 per treatment group. a–e Means in a column without a common letter differ (*p* < 0.05), as analysed by two-way ANOVA and the Duncan test. ^1^ G × C = Gelling × Concentration interaction effect.

**Table 4:** Proximate compositions of different gel-based feeds.

Parameters	Concentration	Moisture	Protein	Lipid	Ash
Pectin	2%	71.6 ± 1.5 ab	36.8 ± 0.4 b	4.7 ± 0.6 bc	10.9 ± 0.2 d
	5%	70.8 ± 1.7 ab	33.8 ± 0.6 cd	7 ± 1 a	8.9 ± 1.4 e
	8%	72.4 ± 7.6 ab	34 ± 0.7 cd	6.3 ± 1.2 ab	9 ± 0.5 e
Calcium lactate	2%	74.5 ± 1.8 a	38.8 ± 0.5 a	4 ± 1 c	12.8 ± 0.03 c
	5%	71.2 ± 1.6 ab	39.9 ± 0.9 a	5.7 ± 0.6 abc	15.9 ± 0.9 a
	8%	67.3 ± 6.1 b	39.6 ± 0.9 a	5.3 ± 1.5 abc	14.4 ± 0.1 b
Starch	2%	75.3 ± 2.6 a	34.5 ± 1.5 c	4.7 ± 1.2 bc	12.7 ± 0.8 c
	5%	72.1 ± 0.1 ab	37.1 ± 0.8 b	4 ± 1 c	7.8 ± 0.04 f
	8%	72.1 ± 0.8 ab	35.9 ± 0.6 b	5.3 ± 0.6 abc	8.4 ± 0.2 ef
Control	0%	75.4 ± 0.5 a	32.5 ± 0.9 d	4.7 ± 1.2 bc	10.2 ± 0.4 d
*p*-value	Gelling	0.400	<0.001	0.030	<0.001
	Conc.	0.130	0.602	0.035	<0.001
	G × C ^1^	0.355	<0.001	0.153	<0.001

Values are means ± SD, *n* = 3 per treatment group. a–f Means in a column without a common letter differ (*p* < 0.05), as analysed by two-way ANOVA and the Duncan test. ^1^ G × C = Gelling × Concentration interaction effect.

**Table 5:** Ingredient composition of experimental gel feed for *Puntius conchonius*.

Feed Composition	(%)	P-2%	P-5%	P-8%	CL-2%	CL-5%	CL-8%	S-2%	S-5%	S-8%	C
Fish muscle (g)	100	80	80	80	80	80	80	80	80	80	80
Corn flour (g)	12.5%	10	10	10	10	10	10	10	10	10	10
Yeast (g)	0.5%	0.8	0.8	0.8	0.8	0.8	0.8	0.8	0.8	0.8	0.8
Vitamin–mineral premix (g)	1.25%	1	1	1	1	1	1	1	1	1	1
Gelling agent (g)	-	1.6	4	6.4	1.6	4	6.4	1.6	4	6.4	0
Salt (g)	2.5%	2	2	2	2	2	2	2	2	2	2

## Data Availability

Not applicable.
